# Comparison of ECMO, IABP and ECMO + IABP in the Postoperative Period in Patients with Postcardiotomy Shock

**DOI:** 10.3390/jcdd11090283

**Published:** 2024-09-08

**Authors:** Cagdas Baran, Evren Ozcinar, Ahmet Kayan, Nur Dikmen, Canan Soykan Baran, Mustafa Bahadir Inan

**Affiliations:** 1 Department of Cardiovascular Surgery, Heart Center, Cebeci Hospitals, Ankara University School of Medicine, 06230 Ankara, Turkey; cagdasbaran@gmail.com (C.B.); nurdikmen@yahoo.com (N.D.); mbahadirinan@gmail.com (M.B.I.); 2 Department of Cardiovascular Surgery, Kirikkale High Specialization Hospital, 71300 Kirikkale, Turkey; dr.ahmet.kayan@gmail.com; 3 Department of Cardiovascular Surgery, Ankara 29 Mayıs Hospital, 06105 Ankara, Turkey; canansykn@hotmail.com

**Keywords:** postcardiotomy shock, ECMO, intra-aortic balloon pump

## Abstract

**Background**: This study aims to assess the outcomes and complications of patients who received veno-arterial extracorporeal membrane oxygenation (VA-ECMO) and intra-aortic balloon pump (IABP) support after cardiac surgery at Ankara University Heart Center between 2000 and 2023. **Methods**: We have carried out a retrospective analysis that included 255 patients. Among them, 98 received IABP, 103 received VA-ECMO, and 54 received both VA-ECMO and IABP. Preoperative and postoperative assessments were carried out, including evaluations of left ventricular function and serum creatinine levels. Primary outcomes included 30-day survival and successful VA-ECMO weaning. Complications such as bleeding, sepsis, liver failure, wound infection, and peripheral ischemia were also assessed. **Results**: The weaning rate from VA-ECMO was significantly higher in the combined VA-ECMO and IABP group (81.4%) compared with the other groups (*p* = 0.004). One-year survival was also higher in the combined group (75.9%) (*p* = 0.002). Complications or renal function did not differ significantly among the groups. The primary indication for mechanical support was coronary artery bypass grafting. **Conclusions**: In conclusion, the combined use of VA-ECMO and IABP therapy led to improved weaning and survival rates without increasing the risk of complications. These findings suggest that a combined approach may be beneficial for selected patients with severe cardiac dysfunction post surgery.

## 1. Introduction

Postcardiotomy shock occurs when patients remain at low output after cardiac surgery, and it has a high mortality rate. Postcardiotomy shock is a severe complication following cardiac surgery, characterized by persistent low cardiac output despite maximal medical therapy. Mechanical circulatory support using veno-arterial extracorporeal membrane oxygenation (VA-ECMO) and intra-aortic balloon pump (IABP) are established treatments for this condition. The efficacy of these modalities, both individually and in combination, has been the subject of various studies. VA-ECMO and IABP have life-saving importance after cardiac surgery in critically ill patients. An intra-aortic balloon pump (IABP) is frequently utilized as the initial stage in the transition to mechanical support and as an assist circulatory device. An IABP is a mechanical circulatory support device designed to improve myocardial oxygen supply and reduce myocardial oxygen demand. It consists of a balloon catheter inserted into the descending aorta. The balloon inflates and deflates in synchrony with the cardiac cycle, which enhances coronary perfusion and reduces the workload on the heart. This device is commonly used in cases of severe heart failure or during high-risk cardiac procedures to stabilize the patient’s condition. Its safety profile, ease of application, and efficacy justify its use. The most important benefit is the reduction in myocardial oxygen demand. Additional benefits include supporting coronary circulation and reducing cardiac workload [1]. VA-ECMO is a form of mechanical circulatory and respiratory support that provides both cardiac and pulmonary assistance. It involves a cannula system that withdraws blood from the venous system, oxygenates it through an artificial membrane lung, and then returns it to the arterial system. Severe cardiac dysfunction is an indication of VA-ECMO in conditions such as myocarditis, myocardial infarction, cardiac trauma, and postcardiotomy syndrome, especially when heart failure is considered to be reversible [2]. VA-ECMO reduces right ventricular preload and also left ventricular (LV) preload by diverting blood from the right side of the heart to a large systemic artery [3]. In certain cases, VA-ECMO can be performed by inserting a cannula into both the femoral vein and femoral artery or by directly placing the arterial cannula into the aorta. However, a potential issue with VA-ECMO is the retrograde flow of blood in the aorta, which can lead to increased left ventricular (LV) afterload. In some cases, intra-aortic balloon pump (IABP) counterpulsation is used alongside VA-ECMO to reduce LV afterload and assist with LV unloading. However, there are conflicting opinions on the combined use of ECMO and IABP. Standard guidelines for the clinical use of ECMO have not yet been defined. Early use of ECMO is preferred, if not contraindicated, to prevent deterioration of vital organs, particularly the brain. Inotropic drugs were also administered as part of the treatment. 

There are not enough studies in the literature on the use of both ECMO and IABP together in postcardiotomy shock. Therefore, this issue is still open to discussion. Current guidelines in postcardiotomy shock suggest that more comprehensive multicenter studies involving more patients should be performed [4]. Since IABP is a less invasive and cheaper treatment method compared with VA-ECMO, we evaluated the use of IABP alone, VA-ECMO alone, and VA-ECMO + IABP in patients in terms of both patient benefit and complications. Our study aims to contribute to this body of knowledge by providing a detailed analysis of outcomes and complications associated with the use of ECMO and IABP in a large cohort of patients post cardiac surgery. By comparing outcomes among patients receiving ECMO only, IABP only, and the combination of both, we seek to offer insights into the effectiveness and safety of these strategies in managing postcardiotomy shock.

## 2. Material and Methods

### 2.1. Patient Selection and Features

Between 2000 and 2023, all patients who used VA-ECMO and IABP after cardiac surgery at Ankara University Heart Center were retrospectively included in the study. A total of 98 patients had IABP, 103 patients had VA-ECMO, and 54 patients had both IABP and ECMO, and these patients were subjected to extensive preoperative operative and postoperative evaluation. LV function was based on preoperative and postoperative assessment by an independent cardiologist. Serum creatinine level was observed in all patients every day preoperatively and postoperatively. This study was approved by the institutional review board and the Ankara University Faculty of Medicine Human Research Ethics Committee (date: 8 January 2024, no. 2023/762). Informed consent was obtained for each patient. Clinical data were collected retrospectively for all patients who received IABP support and VA-ECMO at Ankara University Heart Center.

Primary outcomes were defined as 30-day survival and successful weaning from VA-ECMO. Secondary outcomes included the rates of complications and one-year survival rates. The primary endpoint of the study was to assess the effectiveness of the combined VA-ECMO and IABP therapy in improving these outcomes compared with VA-ECMO or IABP alone.

The decision for VA-ECMO was made by the surgeon in the operating theatre or intensive care unit in patients who could not come off cardiopulmonary bypass or whose heart failure persisted despite maximum inotropic support. The decision for IABP implantation was made by the surgeon in the preoperative or postoperative period in patients with severe left ventricular dysfunction. In deciding whether to use ECMO alone, IABP alone, or a combination of both, several factors were considered, including institutional protocols, technical capabilities, and patient-specific needs. Local guidelines often recommend starting with IABP due to its less invasive nature and escalating to ECMO if necessary. Additionally, the availability of equipment and financial constraints played a role in the decision-making process. Patient-specific factors, such as the severity of their condition and response to initial treatments, also influenced whether a combination therapy was employed. The decision to place an intra-aortic balloon pump (IABP) was guided by several hemodynamic parameters and clinical indicators. Typically, IABP was considered when patients exhibited persistent low cardiac output (usually defined as a cardiac index < 2.0 L/min/m^2^) despite receiving optimal medical management, including fluids and inotropic agents. Severe left ventricular dysfunction, as indicated by a markedly reduced left ventricular ejection fraction (LVEF), was another key factor influencing the decision. The placement of IABP was aimed at improving coronary perfusion and reducing left ventricular afterload, thereby assisting in hemodynamic stabilization. However, exact hemodynamic thresholds for IABP placement varied among patients based on their individual clinical status and response to treatment.

In our study, we specifically employed femoral and jugular cannulation techniques for ECMO support. We did not utilize axillary or aortic cannulation approaches.

Femoral Cannulation: For most patients, ECMO was initiated using femoral cannulation. This involves inserting cannulae into the femoral artery and vein. The femoral approach was chosen due to its relative ease of access and its effectiveness in providing adequate support for the majority of our patients.

Jugular Cannulation: In addition to femoral cannulation, jugular cannulation was employed in cases where additional venous access was required, or when femoral access alone was insufficient. Jugular cannulation was typically used in combination with femoral arterial cannulation to enhance venous drainage and improve overall hemodynamic support.

Exclusion of Axillary and Aortic Cannulation: We did not use axillary or aortic cannulation methods in our patient cohort. The choice to exclude these techniques was based on our institutional protocols, the specific needs of the patient population, and the clinical indications for support. Axillary cannulation was not employed due to its increased complexity and the availability of effective alternatives within our practice. Aortic cannulation was not used, as it was not indicated in our cases, and femoral and jugular approaches were sufficient for the required level of circulatory support.

The timing of IABP and ECMO insertion in our study was influenced by the clinical judgment of the attending surgeons, which varied across the extensive study period from 2000 to 2023. This variability reflects the evolution of clinical practices and protocols over time, as well as differences in individual surgeon preferences and patient conditions. We acknowledge that this wide time frame and surgeon discretion may introduce variability in treatment approaches, which could impact the comparability of outcomes. Despite these challenges, we aimed to capture a broad spectrum of clinical experiences and outcomes associated with the use of these devices. The timing of device insertion varied depending on the patient’s response to initial management and the progression of their condition.

### 2.2. Intra-Aortic Balloon Pump (IABP) Management

The IABP may be considered either before or after surgery for patients experiencing cardiogenic shock that fails to improve with fluids or vasopressors. In these critically ill patients, IABP was established prior to VA-ECMO support. A 7.5-F, 40-mL balloon Percor STAT-DL catheter (Datascope Corp, Fairfield, NJ, USA) was used in all cases, inserted through the femoral artery via a percutaneous approach. The distal end of the balloon catheter was positioned in the descending thoracic aorta below the left subclavian artery. The catheter position was confirmed by chest X-ray. The balloon is inflated upon detection of the R wave on the electrocardiogram (ECG). Inflation occurs just before the diastolic notch of the arterial pressure waveform, and deflation occurs prior to ventricular systole. The ECG-triggered IABP was set to 1:1 assist mode with 100% balloon augmentation during VA-ECMO support.

### 2.3. ECMO Management

ECMO support was initiated for patients with persistently low cardiac output despite IABP support and high doses of inotropic agents such as dobutamine, dopamine, and epinephrine. The ECMO circuit comprised a centrifugal pump console, a membrane oxygenator, an integrated heart exchanger with Quadrox D (Maquet, Jostra, Hirrlingen, Germany), an oxygen/air mixer, and an oximetry monitor. All components were coated with heparin. In some patients, ECMO cannulae (Biomedicus, Medtronic; Minneapolis, MN, USA) were inserted through the femoral artery and femoral vein via a surgical incision. In other instances, ECMO cannulae were inserted percutaneously into the femoral artery and femoral vein. A small cannula in the superficial femoral artery was consistently used to maintain arterial flow to the leg.

To commence VA-ECMO, the flow rate in the circuit was gradually increased over several minutes up to 3.5 L/min. This was aimed at maintaining a mixed venous oxygen saturation (SvO_2_) of 70% and a systolic blood pressure between 80 and 100 mm Hg. With full VA-ECMO support, intravenous inotropic support was reduced, and a protective lung ventilation strategy was implemented to facilitate myocardial and pulmonary recovery. During VA-ECMO support, continuous intravenous heparin was administered, maintaining an activated clotting time between 180 and 220 s.

Once the patient achieved hemodynamic stability with minimal inotropic support, the VA-ECMO support was discontinued. Blood gas analyses were conducted every 2 h while the patient was on VA-ECMO support. Routine blood counts and blood chemistry were analyzed every 24 h. The patient’s hematocrit and platelet values were closely monitored, and replacement was performed as necessary. Cardiac function and transthoracic echocardiography were monitored regularly during ECMO flow reduction. Peripheral cannulae were removed in the operating theatre with the repair of the vessels under direct visualization. Patients under both ECMO and IABP support were followed more closely for weaning because of the higher risk of complications.

The choice between percutaneous and cut-down techniques for ECMO cannula insertion was influenced by several factors, including patient anatomy, the urgency of the situation, and the surgical team’s experience.

Percutaneous Cannulation: Percutaneous cannulation was preferred in cases where a minimally invasive approach was feasible and when rapid initiation of ECMO support was necessary. This technique involves inserting the cannulae through a puncture site in the femoral artery and vein, typically using ultrasound guidance to enhance accuracy and reduce complications. The advantages of percutaneous cannulation include reduced surgical trauma, shorter procedure time, and quicker patient recovery. It is particularly suitable for patients with stable hemodynamic conditions where immediate access is critical.

Cut-Down Cannulation: In contrast, cut-down cannulation was employed when percutaneous access was deemed difficult or if there was a need for a more controlled placement of the cannulae. This technique involves making a surgical incision to directly access the femoral vessels, which allows for better visualization and placement of the cannulae. Cut-down cannulation was used in cases where percutaneous attempts failed, in patients with complex anatomical variations, or when the patient’s clinical condition necessitated a more secure and durable cannulation approach. Although more invasive, the cut-down technique can offer better control and reduce the risk of complications such as vessel injury or cannula displacement.

In our study, the decision to use either technique was made based on individual patient factors, including the urgency of ECMO support, vessel anatomy, and the surgeon’s preference and expertise. Both methods were performed with meticulous care to minimize complications and ensure optimal ECMO support.

ECMO Weaning Criteria:

The decision to wean patients from ECMO support was based on several hemodynamic and clinical parameters, which included:

Left Ventricular Ejection Fraction (LVEF): A stable LVEF of ≥30% for at least 24 h was one of the criteria for weaning consideration.

Left Ventricular Outflow Tract Velocity-Time Integral (LVOT VTI): Weaning from ECMO was considered when the LVOT VTI was consistently above 12 cm, indicating adequate left ventricular function and outflow.

Hemodynamic Stability: Patients had to demonstrate stable hemodynamics, including a systolic blood pressure of ≥90 mmHg without the need for high-dose inotropic support.

Cardiac Output (CO): A cardiac output greater than 3.0 L/min, with minimal or no inotropic support, was required for weaning.

Lactate Levels: Lactate levels needed to be normalized or trending downward (≤2 mmol/L) for a period of 24 h.

Oxygenation and Ventilation: Adequate oxygenation and ventilation parameters were required, including a PaO_2_/FiO_2_ ratio > 200 and minimal ventilatory support.

IABP Weaning Criteria:

Specific criteria for IABP weaning were as follows:

Hemodynamic Improvement: Significant improvement in hemodynamics, including a reduction in the requirement for vasopressors and inotropic support.

Reduction in Symptoms: Relief from symptoms of cardiogenic shock, such as reduced symptoms of pulmonary congestion and improved organ perfusion.

Stable Hemodynamics: A stable mean arterial pressure of ≥65 mmHg and a cardiac index ≥ 2.0 L/min/m^2^ without the need for IABP support.

Myocardial Recovery: Evidence of myocardial recovery, including an improvement in LVEF and a reduction in symptoms of heart failure.

These parameters were closely monitored and assessed daily to ensure that the patient’s condition was appropriate for weaning from ECMO and IABP support.

In the cohort of patients receiving both ECMO and IABP support, the timing of insertion and withdrawal of these devices was as follows:

Insertion Timing:

Intra-Aortic Balloon Pump (IABP) was typically inserted prior to the initiation of ECMO support. The standard approach was to place the IABP first to stabilize hemodynamics and optimize coronary perfusion.

ECMO was then initiated in cases where IABP alone was insufficient to achieve adequate hemodynamic support. The decision to start ECMO was based on persistent low cardiac output despite IABP support and high-dose inotropic agents.

Withdrawal Timing:

The weaning and withdrawal process began with the ECMO support. ECMO was gradually reduced based on improvement in cardiac function and hemodynamic stability.

Once the patient demonstrated stable hemodynamics and adequate myocardial function with reduced or minimal inotropic support, the ECMO was removed first.

Following ECMO removal, the IABP was then withdrawn. The decision to remove the IABP was made based on a clinical assessment of the patient’s hemodynamic status and recovery of myocardial function.

In all patients, 30-day survival was primarily evaluated. VA-ECMO weaning with myocardial recovery was also evaluated. Routine LDH (Lactate dehydrogenase) values were obtained from the patients. Postoperative creatinine increase was evaluated by daily creatinine values, and CRRT was established in some patients. All patients were evaluated for bleeding, stroke, liver failure, wound infection, sepsis, and peripheral ischemia.

### 2.4. Statistical Analysis

Statistical analysis was performed using the SPSS V23 package suitable for the purpose. In addition to descriptive statistical methods (Mean, Standard deviation), the ANOVA test, Mann–Whitney U test, and chi-square tests were used for intragroup comparisons of parameters showing normal distribution in the comparison of quantitative data. The results were evaluated at a 95% confidence interval, and significance was evaluated at *p* < 0.05.

## 3. Results

As shown in Table 1, we identified and retrospectively analyzed 255 patients in whom ECMO and IABP were established in our institution between 2000 and 2023. Patients in whom we performed heart transplantation and LVAD (Left Ventricular Assist Device) operation were excluded. In terms of preoperative characteristics such as diabetes, hypertension, and BMI (Body Mass Index), similar rates were found in all three patient groups. There was no statistically significant difference in all three groups. A total of 173 of these patients, i.e., 67.8%, were male, and there was no statistically significant difference in the male sex ratio in all three groups. Those with peripheral arterial disease in all three patient groups are shown in Table 1 (*p* = 0.456). When serum lactate levels and D-dimer levels were compared in the patient groups, similar results were observed and were not statistically significant (Table 1). As shown in Table 2, preoperative LV ejection fraction did not differ significantly between the groups. The main pathology requiring VA-ECMO or IABP setup was the CABG (coronary artery bypass grafting) operation. The total number of patients who underwent CABG was 144, representing 56.4% of all patients. Regarding preoperative characteristics, among the patients who underwent coronary artery bypass grafting (CABG), 40% had non-ST elevation myocardial infarction (NSTEMI), 25% had ST elevation myocardial infarction (STEMI), and the remainder had other indications such as unstable angina or cardiogenic shock. Additionally, 15% of the surgeries were urgent, reflecting the severity of the patients’ conditions at the time of operation. The other procedures were valve operations, dissections, and combined operations, and there was no statistically significant difference between all surgical procedures in the three groups. There was no significant difference between aortic cross-clamping time (*p* = 0.433) and CPB time (*p* = 0.759) in all three patient groups.

When we compared the weaning status of patients with VA-ECMO and IABP, the weaning rate of patients with both VA-ECMO and IABP was 44 (81.4%), which was more successful and statistically significant compared with the other two groups (*p* = 0.004) (Table 3). When comparing the duration of support with ECMO, IABP, and ECMO + IABP devices among patients in days, the difference between the three groups was statistically insignificant (*p* > 0.05). When calculating these durations, the time until weaning from both devices was included for patients receiving ECMO + IABP. Additionally, the mortality rates during support were examined in all three patient groups. Among patients receiving ECMO + IABP, only five patients died while on support. This finding is statistically significant compared with the other two groups (*p* = 0.011). Again, the 1-year survival of the patients was 41 (75.9%) in patients with both VA-ECMO and IABP, which was better than the other two groups and statistically significant (*p* = 0.002). The reason for this is that in these patient groups, IABP additionally increases coronary perfusion in addition to VA-ECMO. The peripheral leg ischemia rates of these invasive procedures in patients did not increase in patients with both ECMO and IABP, because all of these patients were closely followed in intensive care in every sense. Other complications such as dialysis liver failure and sepsis stroke were similar in all three patient groups and were not statistically significant.

Over the span of the study, our approach to using ECMO and IABP evolved in response to changing guidelines and findings from the Shock II trial. Initially, IABP was more commonly used as a standalone treatment or in conjunction with ECMO. However, as evidence from the Shock II trial and other studies emerged, emphasizing ECMO’s benefits, there was a noticeable shift. We observed a decline in the use of IABP alone and an increase in the use of ECMO, with or without IABP, as a strategy for hemodynamic support and myocardial unloading. This shift reflects a more tailored approach based on the evolving evidence and clinical practice guidelines.

## 4. Discussion

In our study, the main indication for ECMO was an inability to wean from cardiopulmonary bypass. In this study, 1-year survival and weaning rates were more successful in patients with ECMO with IABP setup than in patients with ECMO only and IABP only. The most important reason for this is that IABP plays an additional role in LV unloading and increasing coronary perfusion. In addition, no increase in complications such as stroke, peripheral vascular disease, liver failure, sepsis, and renal failure was observed as a result of the installation of both devices. Shotaro Aso et al. analyzed 1650 patients and found both improved survival and successful weaning from ECMO when IABP was added [5]. Djordjevic et al. found higher weaning rates in ECMO patients after postcardiotomy shock when an additional IABP was used, but they did not find any change in survival [6]. Smedira et al. included 202 patients who underwent ECMO in postcardiotomy shock in their study in 2001 and showed that the lack of simultaneous IABP was a determinant of mortality [7].

Our study’s findings offer significant insights into the outcomes and complications associated with the use of ECMO and IABP in a postoperative cardiac surgery setting. One of the main things we looked at was how long patients survived after 30 days and how successful we were at taking them off ECMO. In our cohort, the weaning rate for patients who received both ECMO and IABP was 81.4%, significantly higher than those who received either ECMO or IABP alone (*p* = 0.004). Similarly, the survival rate after 1 year for the combined group was 75.9%, significantly better than the other two groups (*p* = 0.002). These results are consistent with recent studies that show better outcomes with combined mechanical support. For example, in the study by van den Brink et al., survival until hospital discharge in the ECMO + IABP group was 100% [8].

Furthermore, our study revealed that the addition of IABP to ECMO did not increase the incidence of complications such as dialysis, neurological, or peripheral leg ischemia. This observation corroborates with findings from Yongnan Li et al., who reported no significant difference in the complication rates between patients receiving ECMO alone and those receiving both ECMO and IABP. The study emphasized that with careful monitoring and management in an intensive care setting, the risks associated with combined mechanical support can be minimized [9].

Renal function, as assessed by serum creatinine levels and the need for continuous renal replacement therapy (CRRT), did not show significant differences across the groups in our study. This result aligns with a study by Lian-Yu Lin et al., which demonstrated that using IABP in conjunction with ECMO did not worsen renal impairment in postoperative cardiac patients. Huang et al. also emphasized the importance of timely intervention and close monitoring as crucial in managing renal function in these patients [10].

Furthermore, the primary pathology requiring ECMO or IABP support in our cohort was coronary artery bypass grafting (CABG), accounting for 56.4% of all cases. This is comparable to the study by Björnsdóttir et al. in which CABG was the leading reason for postoperative ECMO and IABP support, accounting for approximately 60% of cases [11]. This consistency points to a common clinical pattern in which CABG patients frequently require advanced circulatory support due to the complexity and severity of their cardiac condition.

In terms of survival rates, our results, showing better outcomes with combined ECMO and IABP support, are supported by a meta-analysis by Zeng et al. in which the dual use of these devices significantly improved survival rates in patients with severe left ventricular dysfunction [12]. They suggested that the synergistic effect of IABP in improving coronary perfusion while ECMO maintained general circulatory support contributed to these improved outcomes. 

The choice between ECMO alone, IABP alone, or the combined use of both modalities was influenced by several factors, including local protocols, technical capabilities, and financial constraints. While the combined use of ECMO and IABP is known to be effective in many cases, the decision to use a single modality was often based on initial clinical assessments, resource availability, and cost considerations. These factors highlight the need for individualized treatment strategies tailored to each patient’s condition and available resources. The combined use of IABP and ECMO aims to optimize hemodynamic support by addressing both myocardial oxygen demand and overall circulatory support. Literature supports this approach in complex cases where patients exhibit high afterload and low cardiac output despite maximal inotropic support [9,13]. IABP helps reduce left ventricular afterload and improves coronary perfusion, while ECMO provides comprehensive circulatory support, making the combination particularly beneficial in severe, refractory cases [14]. This strategy is often employed when there is a need to balance the support for both the heart and the systemic circulation, especially in patients with multiorgan dysfunction or extreme hemodynamic instability.

Our study underscores the effectiveness of combining ECMO and IABP for patients with postcardiotomy shock, aligning with existing clinical literature that supports this approach. Notably, our findings are consistent with several studies demonstrating that combined ECMO and IABP support improves hemodynamic parameters and survival rates compared with using either modality alone [9,13].

In addition to the clinical evidence, experimental studies provide valuable insights into the mechanisms underlying the efficacy of these therapies. For example, the research by Djordjevic et al. offers important experimental perspectives on the interaction between mechanical circulatory support devices and myocardial function [14]. This study used animal models to investigate the impact of combined ECMO and IABP support on myocardial tissue and systemic hemodynamics. The authors observed that the combination therapy not only improved myocardial perfusion but also reduced cardiac workload more effectively than either device used in isolation. This experimental evidence supports the clinical observations of improved outcomes with combined therapy and provides a mechanistic basis for these benefits.

Moreover, Djordjevic et al.’s work highlights the potential for experimental research to identify optimal settings and configurations for mechanical support devices. Their findings suggest that tailored device settings can enhance the efficacy of combined ECMO and IABP support, which has direct implications for clinical practice [14]. This aligns with our study’s results, where patient outcomes were significantly improved with the combined approach, particularly in those with severe cardiogenic shock.

The integration of experimental research with clinical findings underscores the complexity of managing postcardiotomy shock and the need for a multifaceted approach. Experimental studies, such as those conducted by Djordjevic et al., are instrumental in elucidating the physiological effects of mechanical support devices and guiding clinical strategies to maximize their effectiveness. Our study complements this body of work by providing empirical data from a large patient cohort, reinforcing the benefits of combined ECMO and IABP therapy while also highlighting the importance of individualized patient management.

In summary, the combination of clinical and experimental evidence supports the use of ECMO and IABP in managing severe postcardiotomy shock. Future research should continue to explore both clinical outcomes and experimental mechanisms to refine treatment protocols and improve patient outcomes. The collaboration between experimental and clinical research will be crucial in advancing our understanding and optimizing mechanical circulatory support strategies.

### Study Limitations

Single-Center Design: Our study is a single-center, retrospective analysis conducted at Ankara University Heart Center. This design limits the generalizability of our findings to other institutions or patient populations. Results from a multicenter study could provide a more comprehensive view of the effectiveness of combined ECMO and IABP therapy across different settings.

Retrospective Nature: Being a retrospective study, our analysis is subject to inherent biases such as selection bias and data recording inconsistencies. The decision to use ECMO, IABP, or their combination was made based on clinical judgment, which could introduce variability in patient selection and treatment protocols.

Variability in Protocols: The treatment protocols for ECMO and IABP, including device settings and weaning strategies, varied among patients. This variability could influence the outcomes and complicate direct comparisons between different groups. Future studies should aim to standardize treatment protocols to reduce variability and enhance the reliability of comparisons.

Lack of Randomization: The lack of randomization in the treatment allocation may introduce selection bias. Patients receiving combined ECMO and IABP therapy were not randomly assigned, which could affect the comparability of the groups and the interpretation of the outcomes.

Short-Term Follow-Up: While our study provides data on 30-day survival and 1-year survival, longer-term follow-up would provide a more comprehensive assessment of the long-term outcomes and potential late complications associated with combined mechanical support.

Limited Data on Long-Term Complications: Our study focused on immediate complications and outcomes. Future research should investigate long-term complications and quality of life post treatment to provide a more complete picture of the impact of combined ECMO and IABP therapy.

In summary, while our study contributes valuable data to the understanding of combined ECMO and IABP therapy, addressing these limitations through multicenter, prospective, and randomized studies will be crucial for validating and expanding upon our findings. Future research should focus on standardizing treatment protocols, extending follow-up periods, and exploring long-term outcomes to further refine patient management strategies and improve clinical outcomes. The variability in the timing of IABP and ECMO insertion, as well as the reliance on surgeon discretion, is a notable limitation of our study. The extensive time frame of our study, spanning over two decades, reflects changes in clinical practices and advances in medical technology. The lack of a standardized protocol for device insertion during this period may introduce variability in treatment approaches and outcomes. Future studies with more recent data and standardized protocols could provide more precise insights into the optimal timing and decision-making processes for the use of IABP and ECMO in postcardiotomy shock.

## 5. Conclusions

In conclusion, our study supports the growing evidence that combined ECMO and IABP therapy may lead to better clinical outcomes in terms of weaning success and long-term survival without increasing the risk of major complications. These findings suggest that in carefully selected patients, the combined use of ECMO and IABP should be considered to optimize postoperative recovery in severe cardiac cases. Future prospective studies are necessary to further refine patient selection criteria and to validate these findings in larger, multicenter cohorts. Our study has a few limitations. Firstly, it is a single-center retrospective study. Additionally, the decision to use ECMO and IABP was made individually by physicians, leading to significant variability among our patients. As such, a multicenter, prospective study is now essential to better assess the effectiveness of ECMO and IABP treatments in patients experiencing postcardiotomy shock.

## Figures and Tables

**Table 1:** Preoperative patient characteristics.

	VA-ECMO (*n* = 103)	VA-ECMO + IABP (*n* = 54)	IABP (*n* = 98)	*p*
Age	63.3 ± 12.2	64.1 ± 11.3	63.9 ± 13.4	0.678
BMI	28.2 ± 4.1	26.4 ± 5.32	28.7 ± 4.65	0.431
Male	65 (63.1%)	37 (68.5%)	71 (72.4%)	0.546
Hypertension	56 (54.3%)	32 (59.2%)	49 (50%)	0.224
Smoker	31 (30.09%)	24 (44,4%)	44 (44.8%)	0.360
Diabetes mellitus	29 (28.1%)	18 (33,3%)	33 (33.6%)	0.583
Creatinin	1.61 ± 1.21	1.56 ± 0.83	1.29 ± 0.92	0.224
EuroScore	7.8 ± 1.9	6.2 ± 2.2	9.2 ± 1.8	0.192
Peripheral arterial disease	7 (6.7%)	4 (7.4%)	6 (6.1%)	0.456
Cardiopulmonary resuscitation	23 (22.3%)	12 (22.2%)	20 (20.4%)	0.391
Serum lactate (mmol/L)	5.34 ± 3.44	6.56 ± 4.1	5.87 ± 3.56	0.54
D-dimers (mg/L)	5.15 ± 2.34	6.77 ± 3.89	5.65 ± 2.8	0.156

Data are presented as *n* (%) or mean ± standard deviation. VA-ECMO, veno-arterial extracorporeal membrane oxygenation; IABP, intra-aortic balloon pump; BMI, body mass index; SOFA, sequential organ failure assessment.

**Table 2:** Types of surgery and operation data.

	VA-ECMO (*n* = 103)	VA-ECMO + IABP (*n* = 54)	IABP (*n* = 98)	*p*
CABG	58 (56.3%)	32 (59.2%)	56 (57.1%)	0.344
Valve replacement	26 (25.2%)	9 (16.6%)	23 (23.4%)	0.433
Aort dissection	5 (4.8%)	2 (3.7%)	7 (7.1%)	0.376
Combined surgery	14 (13.5%)	11 (20.3%)	12 (12.2%)	0.223
LVEF >50%	12 (11.6%)	13 (24.07%)	23 (23.4%)	0.433
LVEF 30%–50%	57 (55.3%)	23 (42.5%)	45 (45.9%)	0.192
LVEF <30%	34 (33%)	18 (33.3%)	30 (30.6%)	0.134
CPB time	03:49 ± 1:45	03:57 ± 1:56	03:55 ± 1:59	0.759
Aortic cross-clamp time	1:59 ± 0:56	1:56 ± 0:57	1:49 ± 0:78	0.433

Data are presented as *n* (%) or mean ± standard deviation.VA-ECMO, veno-arterial extracorporeal membrane oxygenation; IABP, intra-aortic balloon pump; CABG, coronary artery bypass grafting; LVEF, left ventricular ejection fraction; CPB, cardiopulmonary bypass.

**Table 3:** Complications and survivals.

	VA-ECMO (*n* = 103)	VA-ECMO + IABP (*n* = 54)	IABP (*n* = 98)	*p*
Stroke	17 (16.5%)	12 (22.2%)	21 (21.4%)	0.533
Sepsis	21 (20.3%)	12 (22.2%)	18 (18.3%)	0.459
Reoperation	34 (33%)	29 (53.7%)	45 (45.9%)	0.659
Dialysis	49 (47.5%)	28 (51.8%)	44 (44.8%)	0.212
Limb ischemia	8 (7.7%)	6 (11.1%)	12 (12.2%)	0.123
Liver failure	21 (20.3%)	11 (20.3%)	18 (18.3%)	0.053
Successful weaning ECMO and IABP	74 (71.8%)	44 (81.4%)	61 (62.2%)	0.004
Time on devices, day	6.4 ± 4.8	7.3 ± 5.8	4.6 ± 4.1	0.245
Survival, 1 year	67 (65.04%)	41 (75.9%)	52 (53.06%)	0.002
Survival, 30 days	83 (80.5%)	47 (87%)	73 (74.4%)	0.063
Death on support	16 (15.5%)	5 (9.2%)	21 (21.4%)	0.011
Alive and Weaned	87 (84.4%)	49 (90.7%)	77 (78.5%)	0.087

Data are presented as *n* (%) or mean ± standard deviation. VA-ECMO, veno-arterial extracorporeal membrane oxygenation; IABP, intra-aortic balloon pump.

## Data Availability

Data are contained within the article.
